# Primary transmission of chronic wasting disease versus scrapie prions from small ruminants to transgenic mice expressing ovine or cervid prion protein

**DOI:** 10.1099/jgv.0.000539

**Published:** 2016-09

**Authors:** Sally A. Madsen-Bouterse, David A. Schneider, Dongyue Zhuang, Rohana P. Dassanayake, Aru Balachandran, Gordon B. Mitchell, Katherine I. O'Rourke

**Affiliations:** ^1^​ Department of Veterinary Microbiology and Pathology, College of Veterinary Medicine, Washington State University, Pullman, WA 99164-7040, USA; ^2^​ Animal Disease Research Unit, Agricultural Research Service, US Department of Agriculture, Pullman, WA 99164-6630, USA; ^3^​ National and OIE Reference Laboratory for Scrapie and CWD, Canadian Food Inspection, Agency, Ottawa Laboratory–Fallowfield, Ottawa, Ontario, Canada

**Keywords:** scrapie, sheep, goat, chronic wasting disease, mouse bioassay

## Abstract

Development of mice expressing either ovine (Tg338) or cervid (TgElk) prion protein (PrP) have aided in characterization of scrapie and chronic wasting disease (CWD), respectively. Experimental inoculation of sheep with CWD prions has demonstrated the potential for interspecies transmission but, infection with CWD versus classical scrapie prions may be difficult to differentiate using validated diagnostic platforms. In this study, mouse bioassay in Tg338 and TgElk was utilized to evaluate transmission of CWD versus scrapie prions from small ruminants. Mice (≥5 per homogenate) were inoculated with brain homogenates from clinically affected sheep or goats with naturally acquired classical scrapie, white-tailed deer with naturally acquired CWD (WTD-CWD) or sheep with experimentally acquired CWD derived from elk (sheep-passaged-CWD). Survival time (time to clinical disease) and attack rates (brain accumulation of protease resistant PrP, PrP^res^) were determined. Inoculation with classical scrapie prions resulted in clinical disease and 100 % attack rates in Tg338, but no clinical disease at endpoint (>300 days post-inoculation, p.i.) and low attack rates (6.8 %) in TgElk. Inoculation with WTD-CWD prions yielded no clinical disease or brain PrP^res^ accumulation in Tg338 at endpoint (>500 days p.i.), but rapid onset of clinical disease (~121 days p.i.) and 100 % attack rate in TgElk. Sheep-passaged-CWD resulted in transmission to both mouse lines with 100 % attack rates at endpoint in Tg338 and an attack rate of ~73 % in TgElk with some culled due to clinical disease. These primary transmission observations demonstrate the potential of bioassay in Tg338 and TgElk to help differentiate possible infection with CWD versus classical scrapie prions in sheep and goats.

## Introduction

Classical scrapie is a naturally occurring transmissible spongiform encephalopathy (TSE) of sheep and goats. Though it has been recognized in some regions of the world for over 250 years (reviewed by Schneider *et al.*, 2008), classical scrapie (herein just ‘scrapie’) was first diagnosed in the USA in 1947 following the import of an infected sheep. In contrast, chronic wasting disease (CWD) is a naturally occurring TSE of cervids (elk, deer and moose) which was first observed in the 1960s in deer held captive in several wildlife facilities in the USA (Williams & Young, 1980; reviewed by Haley & Hoover, 2015). Both scrapie and CWD are transmissible, infectious, neurodegenerative diseases for which there are no treatments. Prions, the novel proteinaceous infectious particles that are believed to be the causative agent of TSEs, induce misfolding of normal cellular prion protein (PrP^C^) to a form that is relatively resistant to protease cleavage (PrP^res^) (Prusiner, 1982; review by Ryou, 2007). Misfolded PrP associated with scrapie disease is often abbreviated PrP^Sc^, whereas in CWD, the misfolded protein may be abbreviated PrP^CWD^. A hallmark of prion diseases is the slow, progressive development of central neuropathology that is accompanied by accumulation of misfolded PrP. After a variably long incubation period, clinical disease often manifests as pruritus, ataxia, weight loss, tremors and changes in behaviour (review by Haley & Hoover, 2015; Konold & Phelan, 2014), but ultimately leads to the demise of the animal.

Animal bioassay is a gold standard technique for the recognition and characterization of different prion types (Beck *et al.*, 2012b; Bruce *et al.*, 2002; Thackray *et al.*, 2012). The use of wild-type inbred strains of mice (e.g. RIII, C57BL and VM) has aided in differentiating types of scrapie prions as measured by incubation and survival times, histopathologic lesion profiles, brain accumulation profiles of PrP^Sc^ and biochemical characterization of PrP^res^ (Beck *et al.*, 2010, 2012a, b; Bruce *et al.*, 2002, 2003; Vulin *et al.*, 2012). In contrast, there have been few observations of CWD transmission to wild-type mice (Lee *et al.*, 2013). To reduce incubation times and number of subpassages for studies of scrapie prions and improve transmission efficiency of CWD prions, transgenic mice were developed to express the ovine or cervid prion protein gene (*PRNP*), respectively (Browning *et al.*, 2004; Cordier *et al.*, 2006; Crozet *et al.*, 2001; LaFauci *et al.*, 2006; Tamgüney *et al.*, 2006; Vilotte *et al.*, 2001). Using transgenic mice expressing ovine *PRNP* (ovinized mice), scrapie incubation times were substantially reduced compared with incubation times in wild-type mice (Crozet *et al.*, 2001; Vilotte *et al.*, 2001) while histopathologic profiles and PrP^res^ biochemical features characteristic of scrapie prion types were maintained (Baron *et al.*, 2004; Chong *et al.*, 2015; Cordier *et al.*, 2006). In addition, ovinized mice have been shown to be permissive to scrapie prions derived from goats with naturally acquired disease (O'Rourke *et al.*, 2012). Several transgenic mouse lines expressing cervid *PRNP* (cervidized mice) have been generated for studies of CWD prions. Cervidized mice have shown reduced incubation times with increased attack rates compared with wild-type mice (Lee *et al.*, 2013) and have proven useful in the discrimination of CWD prion types isolated from different species [mule deer versus white-tailed deer (WTD) versus elk] (Browning *et al.*, 2004; LaFauci *et al.*, 2006; Tamgüney *et al.*, 2006). Thus, ovinized and cervidized mice have been established as appropriate mouse bioassay models for the study of scrapie and CWD prions, respectively.

As countries work diligently to eradicate scrapie in sheep and goats, it is important to develop tools that differentiate disease resulting from novel versus known sources of prions in an effort to identify TSE reservoirs that could lead to disease re-emergence, especially in regions where other TSEs naturally exist. A potentially novel source to small ruminants would be from natural exposure to CWD prions. CWD continues to increase in both prevalence and geographic distribution in North America (reviewed by Haley & Hoover, 2015); the longest known presence in the USA is in the states of CO and WY (Williams & Young, 1980). Sheep are susceptible to CWD prions when inoculated intracerebrally with brain homogenate from mule deer with CWD, but the attack rate is low and *PRNP* genotype of the recipient sheep may contribute to susceptibility (Hamir *et al.*, 2006). It is currently unknown if sheep naturally exposed to CWD prions will readily become infected or if the resulting TSE would appear different (clinically and/or diagnostically) from classical scrapie in sheep and goats. Transgenic mice have been used to examine the potential for TSEs to cross the species barrier (Beck *et al.*, 2012a; Tamgüney *et al.*, 2006). This study evaluates primary transmission of scrapie prions from goats or sheep and CWD prions from WTD to both cervidized and ovinized transgenic mice (TgElk and Tg338, respectively). Furthermore, mouse bioassay is used to assess transmission of CWD prions to these mice following primary experimental passage through sheep.

## Results

### PrP^res^ characteristics in donor inocula

**Fig. 1.**
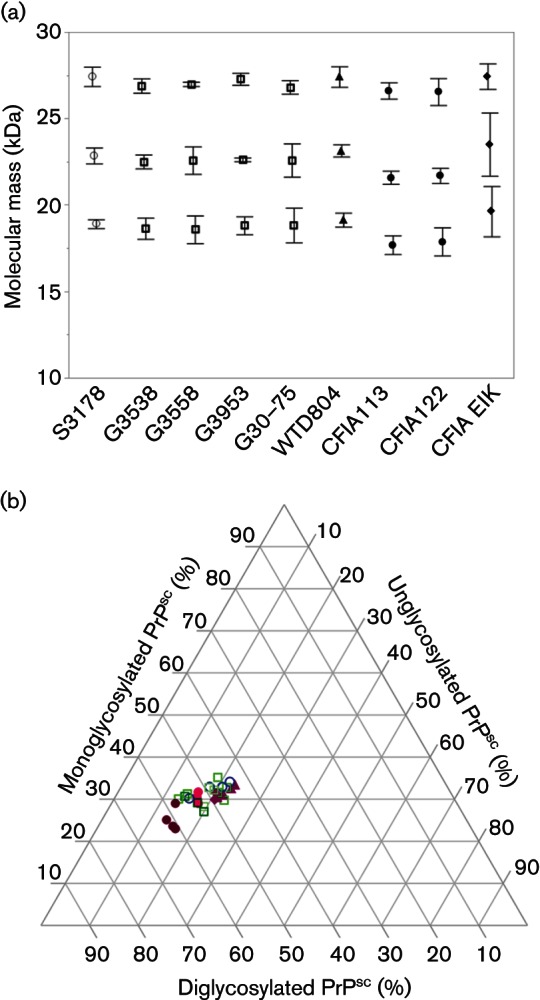
Characterization of PrP^res ^in scrapie and CWD brain homogenate inocula. Shown are the (a) molecular masses and (b) relative densities of di-, mono- and unglycosylated PrP^res^. Data were derived from three or four independent Western blots performed using mAb F99/97.6.1. Least squares means and 95 % confidence intervals are plotted in (a). Symbol representations for TSEs and donor animals: open circle, scrapie from sheep S3178; open square, scrapie from goats G3538, G3558, G3953 and G30-75; closed triangle, CWD from white-tailed deer WTD804; closed circle, primary passage of CWD from CFIA Elk in sheep CFIA113 and CFIA122; closed diamond, CWD from pooled elk homogenate ‘CFIA Elk’.

Brain homogenates from goats (G3538, G3558, G3953 and G30-75), sheep (S3178) and WTD (WTD804) with naturally acquired TSEs (scrapie in goats and sheep; CWD in deer) were assessed for variation in PrP^res^ molecular mass and glycoform ratios by Western blotting. As expected, some animal-to-animal variation was observed. Molecular mass of unglycosylated PrP^res^ (lowest band, Fig. 1a) from WTD804 was ~0.5 kDa larger (*P*<0.05) than PrP^res^ from G3538 and G3558, but not PrP^res^ from G3953, G30-75 or S3178. Unglycosylated PrP^res^ from brains of sheep (CFIA113 and CFIA122) experimentally inoculated with a pooled homogenate of brain and lymph node from elk with CWD was ~1 kDa smaller (*P*<0.05) compared with PrP^res^ from all animals with naturally acquired scrapie or CWD. Molecular mass of unglycosylated PrP^res^ was also compared between the experimentally inoculated sheep and the pooled elk homogenate (CFIA Elk) the sheep were inoculated with. Unglycosylated PrP^res^ from CFIA113 and CFIA122 was ~1.75 kDa smaller (*P*<0.05) than PrP^res^ from CFIA Elk. The molecular mass of unglycosylated PrP^res^ from CFIA Elk was also ~0.75 kDa larger (*P*<0.05) than PrP^res^ from goats with naturally acquired scrapie, but not significantly different from PrP^res^ in the sheep or WTD homogenates tested. Using a three-axis plot to visualize glycoform ratios, diglycosylated PrP^res^ appeared to be the most abundant, and unglycosylated PrP^res^ the least abundant in all homogenates (Fig. 1b).

The relative level of misfolded PrP in each donor brain homogenate used for mouse inoculations was assessed by enzyme immunoassay (EIA) and expressed as the equivalent starting wet tissue weight of the endpoint serial dilution. Levels of misfolded PrP were similar for inocula prepared from donor animals G3953, WTD804 and CFIA113 with each at ~6 µg starting wet tissue weight. The last serial dilution that yielded a detectable EIA signal was equivalent to approximately 94 µg for CFIA122. The relative levels of misfolded PrP in the inocula from other donors were similarly determined and previously reported as follows (O'Rourke *et al.*, 2012): ~23 µg starting wet tissue weight for G3558, 46 µg for G3538, 93 µg for G30-75 and 186 µg for S3178.

### Transmission of classical scrapie to Tg338 and TgElk mice

Transgenic mice expressing ovine *PRNP* (Tg338) or cervid *PRNP* (TgElk) underwent intracerebral inoculation with brain homogenate from goats or a sheep with naturally acquired scrapie (G3538, G3558, G3953, G30-75 or S3178) and were monitored for the development of clinical signs associated with TSE disease (Bruce *et al.*, 2004). Mice were culled at clinical disease, for intercurrent disease, or within an endpoint period chosen to limit the occurrence of common age-related health issues (e.g. penile prolapse and chronic conjunctivitis). The endpoint period for Tg338 mice was 500–600 days post-inoculation (p.i.). In comparison to Tg338 mice, TgElk mice are short-lived and required an endpoint cull period of 300–375 days p.i. TgElk mice also demonstrate hyperexcitability at baseline; thus, this behaviour was not considered a clinical sign of TSE disease in this line of mice.

**Fig. 2.**
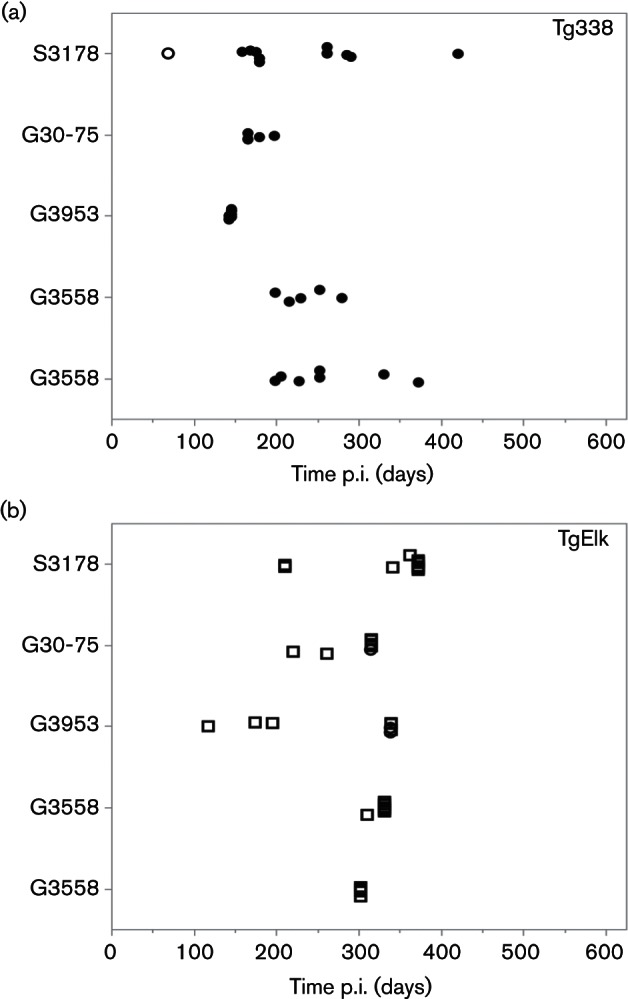
Transgenic mouse bioassay of scrapie. Shown are the outcomes following intracerebral inoculation of Tg338 (a) or TgElk (b) mice with scrapie prions derived from sheep S3178 and goats G3538, G3558, G3953 and G30-75. Closed circles, mice culled at the onset of clinical disease and PrP^res ^detected in the brain; open circles, preclinical mice (culled due to intercurrent health issues or during the endpoint period with no clinical signs but PrP^res^ detected in the brain); open squares, mice with no detectable PrP^res^ in the brain at the time of cull. Censored data are included. Though variable in survival time, nearly all Tg338 mice inoculated with sheep brain homogenate harbouring classical scrapie prions developed clinical disease, whereas none of the TgElk mice similarly inoculated developed clinical disease.

**Fig. 3.**
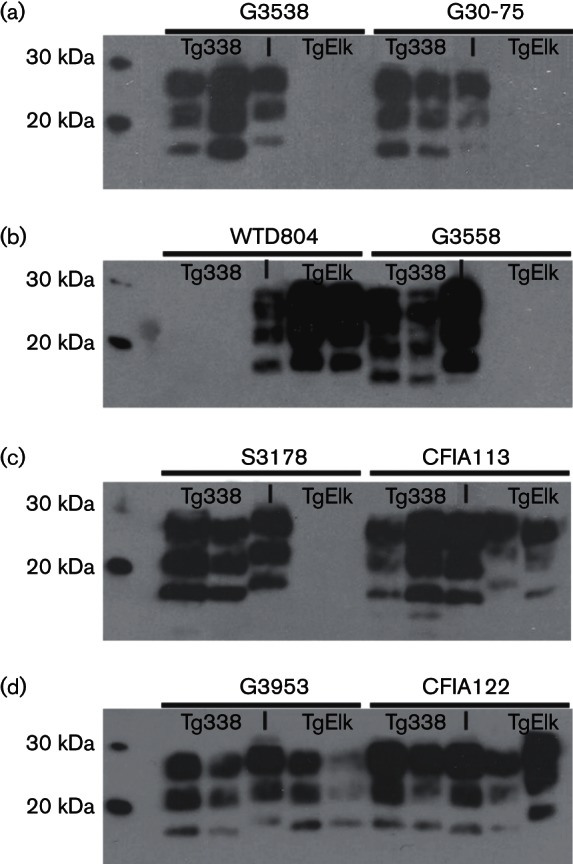
PrP^res^ in inocula, Tg338 and TgElk brain homogenate. Representative Western blots with mAb F99/97.6.1 of brain homogenates from original inocula (I) and two recipients per mouse line (Tg338 or TgElk) for each inocula following PK treatment and precipitation with sodium phosphotungstic acid (NaPTA). All samples were prepared as 10 % homogenates (w/v) in PBS. (a) Brain homogenates from goats with naturally acquired scrapie (G3538 and G30-75) and recipient Tg338 and TgElk mice. (b) Brain homogenate from a white-tailed deer with naturally acquired CWD (WTD804) and recipient Tg338 and TgElk mice; brain homogenate from goat (G3558) with naturally acquired scrapie and recipient Tg338 and TgElk mice. (c) Brain homogenate from sheep (S3178) with naturally acquired scrapie and recipient Tg338 and TgElk mice; brain homogenate from CFIA113 (sheep-passaged-CWD) and recipient Tg338 and TgElk mice. (d) Brain homogenate from goat (G3953) with naturally acquired scrapie and recipient Tg338 and TgElk; brain homogenate from CFIA122 (sheep-passaged-CWD) and recipient Tg338 and TgElk mice.

Tg338 mice inoculated with scrapie prions were culled with clinical signs such as ataxia, extreme lethargy, weight loss, hunched stance or gait, tremors, clenched paws or hyperexcitability. The onset of clinical disease and attack rates for Tg338 mice are shown in Fig. 2(a), and outcomes are summarized in Table 1. Most mice were culled between 143 and 300 days p.i. with additional mice culled at 331, 373 (both in G3538 group) and 421 days p.i. (S3178 group). The accumulation of PrP^res^ in the brain was assessed in Tg338 by Western blot (WB) analysis to determine attack rates (Table 1). All Tg338 mice culled due to clinical disease were positive for PrP^res^ in the brain (see representative WBs in Fig. 3) as was a mouse culled due to intercurrent health issues at an earlier time point (69 days p.i.). The molecular mass of PrP^res^ from Tg338 inoculated with classical scrapie prions showed a small reduction (1–2 kDa) compared with the original inoculum. This reduction was previously demonstrated with mouse bioassay of brain homogenate from S3178, G3538, G3558 and G30-75 (O'Rourke *et al.*, 2012), and was found to be due to an altered proteinase K (PK) cleavage site in PrP^res^ that accumulates in the brain of inoculated Tg338 as compared to that present in the original sheep inoculum.

**Table 1.** Transmission of classical scrapie from sheep and goats with naturally acquired clinical disease to ovinized (Tg338) and cervidized (TgElk) mice

Mouse	Inoculum*	Mice culled at onset of clinical disease /total inoculated	Median survival time (95 % CL†)	Attack rate in mice‡
Tg338	S3178	11/12	262 (169, 286)	12 (1)/12
	G3538	7/7	253 (199, 331)	7/7
	G3558	5/5	230 (199, 280)	5/5
	G3953	8/8	146 (143, 146)	8/8
	G30-75	5/5	166 (166, 198)	5/5
TgElk	S3178	0/11	>210 (NE)	0/11
	G3538	0/5	>302 (NE)	0/5
	G3558	0/9	>310 (NE)	0/9
	G3953	0/9	>117 (NE)	2 (1)/9
	G30-75	0/10	>220 (NE)	1/10

*S, Sheep; G, goat.

†95 % lower and upper confidence limits (LCL, UCL): In the case of right censored survival analysis, lower confidence limits are left bounded, whereas upper confidence limits are unbounded. NE, Non-estimable due to insufficient group sample size for which survival time could be measured.

‡Number of mice with detectable protease resistant prion protein (PrP^res^) in the brain at the time of cull/number of mice tested. All mouse brains were tested by standard (direct lysis) Western blot (WB); brain tissue negative by standard WB was tested using phosphotungstic acid (PTA-WB). The number in parenthesis indicates the number of brains PrP^res^ positive by PTA-WB which is included in the total number positive.

The onset of clinical disease and attack rates in TgElk mice inoculated with brain homogenate from goats or sheep with naturally acquired classical scrapie are shown in Fig. 2(b) and outcomes summarized in Table 1. Clinical signs associated with the onset of TSE disease were not observed before the assay endpoint. All TgElk mice were assessed for brain PrP^res^ accumulation to determine attack rates in the absence of clinical signs (see representative WBs in Fig. 3). Brain PrP^res^ was detected in 3 of 44 mice; the positive mouse culled at 315 days p.i. was inoculated with brain homogenate from G30-75, whereas two mice culled at 339 days p.i. were inoculated with brain homogenate from G3953. Thus, classical scrapie prions transmitted efficiently to Tg338 with near 100 % onset of clinical disease and 100 % attack rates. However, transmission was inefficient in TgElk with no onset of clinical disease prior to the endpoint period and low (~6.8 %) attack rates.

### Transmission of CWD to Tg338 and TgElk mice

**Fig. 4.**
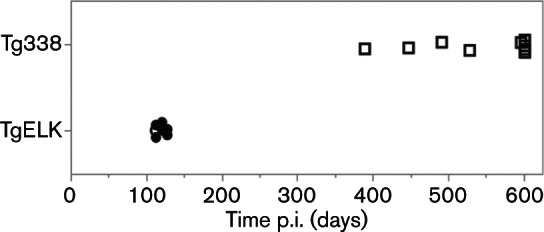
Transgenic mouse bioassay of CWD in Tg338 and TgElk. Shown are the outcomes for the first intracerebral inoculation of mice with CWD derived from white-tailed deer (WTD804). Closed circles, mice culled at the onset of clinical disease and PrP^res ^detected in the brain; open circles, preclinical mice (culled due to intercurrent health issues, PrP^res^ detected in the brain); open squares, mice with no detectable PrP^res^ in the brain at the time of cull. Censored data are included. Tg338 inoculated with white-tailed deer brain homogenate harbouring CWD prions did not develop clinical disease and had no detectable PrP^res^ in the brain, whereas nearly all TgElk mice similarly inoculated were culled at onset of clinical disease and all had detectable PrP^res^ in the brain.

Concurrent with inoculation of scrapie prions, Tg338 and TgElk mice underwent intracerebral inoculation with a pooled brain homogenate from WTD with naturally acquired CWD (WTD804). The onset of clinical disease and attack rates in mice are shown in Fig. 4 and outcomes summarized in Table 2. Tg338 mice inoculated with WTD804 did not demonstrate clinical signs associated with TSE disease. Mice were culled either in the endpoint period or due to intercurrent health issues; brain PrP^res^ accumulation was not detected (see representative WB in Fig. 3). Inoculation of TgElk with WTD804 resulted in the onset of clinical disease. Mice displayed clinical signs such as ataxia, extreme lethargy, weight loss, hunched stance or gait, circling, tail or hind-end mutilation of self or others and extreme obesity to the point of inhibiting movement. All TgElk inoculated with WTD804 were culled by 128 days p.i. and were positive for PrP^res^ in the brain (see representative WB in Fig. 3). Thus, CWD prions were transmitted efficiently to TgElk mice with no evidence of transmission to Tg338.

**Table 2.** Transmission of CWD from white-tailed deer with naturally acquired clinical disease or experimentally inoculated sheep to ovinized (Tg338) and cervidized (TgElk) mice

Mouse	Inoculum*	Mice culled at onset of clinical disease† /total inoculated	Median survival time (95 % CL‡)	Attack rate in mice§
Tg338	WTD804	0/10	>389 (NE)	0/10
	CFIA113	0/9	>499 (NE)	9 (6)/9
	CFIA122	0/9	>499 (NE)	9 (3)/9
TgElk	WTD804	9 (1)/10	121 (113, 128)	10/10
	CFIA113	2 (7)/12	>290 (290, NE)	10 (1)/12
	CFIA122	5/10	>261 (261, NE)	6 (1)/10

*WTD, White-tailed deer; CFIA, experimental intracranial inoculation of sheep with pooled brain and lymph node homogenate from elk with CWD (see Methods for more detail).

†Number of mice culled at the onset of clinical disease/number of mice inoculated. The number in parenthesis indicates additional mice with suspect clinical signs that also had intercurrent health issues at the time of cull. These mice were censored for survival analysis.

‡95  % lower and upper confidence limits (LCL, UCL): In the case of right censored survival analysis, lower confidence limits are left bounded, whereas upper confidence limits are unbounded. NE, Non-estimable due to insufficient group sample size for which survival time could be measured.

§Number of mice with detectable protease resistant prion protein (PrP^res^) in the brain at the time of cull/number of mice tested. All mouse brains were tested by standard (direct lysis) Western blot (WB); brain tissue negative by standard WB was tested using PTA-WB. The number in parenthesis indicates brains PrP^res^ positive by PTA-WB which is included in the total number positive.

### Transmission of CWD to Tg338 and TgElk following primary passage in sheep

**Fig. 5.**
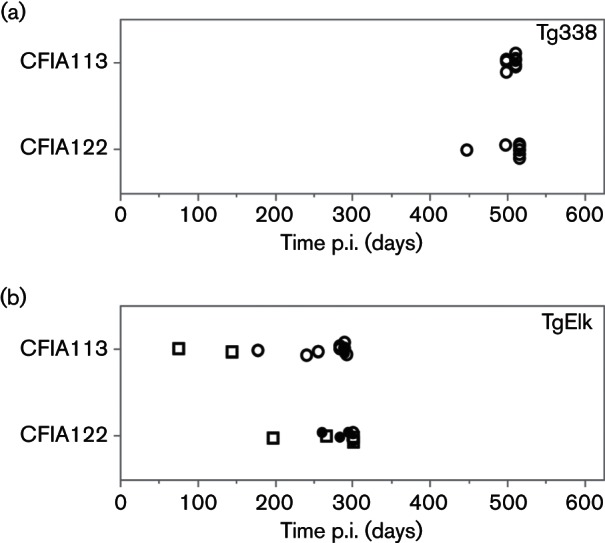
Transgenic mouse bioassay of CWD derived from experimentally infected sheep. Shown are the outcomes of intracerebral inoculation of Tg338 (a) or TgElk (b) mice with CWD prions derived for experimental infections of sheep CFIA113 and CFIA122. Closed circles, mice culled at the onset of clinical disease and PrP^res ^detected in the brain; open circles, preclinical mice (culled due to intercurrent health issues or during the endpoint period with no clinical signs, but PrP^res^ detected in the brain); open squares, mice with no detectable PrP^res^ in the brain at the time of cull. Censored data are included. All Tg338 mice inoculated with sheep brain homogenate harbouring CWD prions had detectable PrP^res^ in the brain when culled in the endpoint period. Some of the similarly inoculated TgElk mice were culled due to the onset of clinical disease just prior to the endpoint period.

To evaluate if transmission of CWD prions was altered following primary passage through sheep, brain homogenates from sheep experimentally inoculated with CWD (CFIA113 and CFIA122) were used to inoculate Tg338 and TgElk mice. The onset of clinical disease and attack rates are shown in Fig. 5 and outcomes are summarized in Table 2. Although Tg338 inoculated with CFIA113 or CFIA122 were non-clinical when culled in the endpoint period, all mice were positive for PrP^res^ in the brain (100 % attack rate; see representative WBs in Fig. 3). Clinical disease was observed near the endpoint period in some TgElk inoculated with CFIA113 or CFIA122. The accumulation of PrP^res^ in the brain was detected in all TgElk that were culled with clinical signs or due to intercurrent health issues in addition to suspected clinical disease. One additional, non-clinical TgElk in each inoculation group was positive for brain PrP^res^, but only when the mouse brain homogenate was first enriched using phosphotungstic acid precipitation (PTA-WB), see Methods for details. Thus, CWD prions following primary passage in sheep transmitted efficiently to both Tg338 and TgElk with 100 and 72.7 % attack rates, respectively.

## Discussion

There are currently no known cases of interspecies transmission of CWD prions to sheep or goats through natural exposure, and transmission resulting from direct inoculation has been relatively inefficient. Nevertheless, the potential for natural exposure of sheep and goats to CWD prions is presumably rising in parallel with the increasing incidence and geographic range of CWD occurring in both free-ranging and farmed cervids in North America (review by Haley & Hoover, 2015). Should interspecies transmission of CWD occur naturally, the ability to differentiate CWD versus scrapie infection in sheep and goats may enhance epidemiological investigations and surveillance efforts attempting to identify novel sources of prion exposure. This study assessed transmission of classical scrapie and CWD prions from their native hosts to transgenic mouse lines expressing the ovine or cervid PrP. Inoculation of transgenic mice with scrapie prions from goats or sheep or CWD prions from WTD resulted in distinct transmission patterns. Scrapie prions transmitted efficiently to Tg338 but not TgElk, whereas CWD prions from a natural host efficiently transmitted to TgElk but not Tg338. Unlike these distinct transmission patterns, efficient transmission of CWD prions following primary passage in sheep was observed in both Tg338 and TgElk mice.

Experimentally transmitted CWD in small ruminants has been difficult to distinguish from natural cases of classical scrapie. In sheep, intracerebral inoculation of CWD prions from mule deer resulted in immunohistochemistry-detected brain and tonsil accumulation patterns and PrP^res^ molecular profiles that were very similar to those observed in classical scrapie (Hamir *et al.*, 2006). In the current study, small variations in molecular mass of brain homogenate PrP^res^ were detected among species (Fig. 1), but the variation observed in those tested did not segregate infection with scrapie versus CWD prions. This agrees with previous reports of variation in PrP^res^ molecular mass (Stack *et al.*, 2004) and no significant variation in glycoform ratios (Race *et al.*, 2002). Further, PrP^res^ from sheep inoculated with elk CWD prions (sheep-passaged-CWD) was significantly different from both scrapie- and CWD-associated PrP^res^ from their native hosts. While these shifts in PrP^res^ molecular mass occur upon primary interspecies transmission, they are likely too small as compared to natural variation of scrapie PrP^res^ to allow robust differentiation of prion type. Thus, consistent with the conclusion of Hamir *et al*. (2006), standard methods will detect, but are not likely to differentiate, primary infections of sheep or goats with CWD prions versus scrapie prions.

The use of Tg338 and TgElk mouse lines is well established in the respective study of scrapie and CWD (Beck *et al.*, 2013; Jeon *et al.*, 2013; LaFauci *et al.*, 2006; Langevin *et al.*, 2011; O'Rourke *et al.*, 2012; Thackray *et al.*, 2011a, b; Tixador *et al.*, 2010; Vilotte *et al.*, 2001). Such studies include the use of Tg338 mice to differentiate strains of classical scrapie prions within sheep (Thackray *et al.*, 2008), to detect mixed infection of sheep with classical and atypical scrapie prions (Chong *et al.*, 2015) and to show the similar transmission phenotypes of classical scrapie prion isolates from sheep and goats (O'Rourke *et al.*, 2012). Similar studies have used cervidized mice, including TgElk, to examine the phenotypes of CWD prions isolated from cervid species (Browning *et al.*, 2004; Duque Velasquez *et al.*, 2015; LaFauci *et al.*, 2006). Few studies have used ovinized and cervidized mice to model interspecies infection of CWD and scrapie. Tg338 intracerebrally inoculated with elk CWD prions resulted in low transmission rates observed in the brain but high transmission rates observed in the spleen suggesting that cross-species transmission could preferentially accumulate in peripheral lymphoid tissues (Béringue *et al.*, 2012). Tamgüney and colleagues found no evidence of CWD transmission to Tg338 mice using inocula derived from WTD, elk or mule deer (Tamgüney *et al.*, 2006) or to a different ovinized mouse using inoculum from WTD (Tamgüney *et al.*, 2009). A similar lack of detectable transmission was observed in brains of Tg338 inoculated with WTD804 in the current study (Fig. 4, Table 2). Studies evaluating susceptibility of cervidized mice to scrapie are also limited. Browning *et al*. (2004) did not observe transmission of mouse-adapted scrapie (RML strain) into one cervidized mouse line. Using a different cervidized mouse line, Tamgüney and colleagues observed transmission of RML scrapie and a European strain of scrapie serially passaged in sheep (SSBP/1), but not a natural scrapie isolate from a North American sheep (Tamgüney *et al.*, 2009). In the current study, a low overall attack rate (~6.8 %) was observed in TgElk inoculated with scrapie prions derived from clinical cases in North America. The evidence of transmission to TgElk, albeit low, could be due to the use of a different cervidized mouse than what was used in previous studies by others. Another possible contributing factor to the low attack rates could be that the homogenates resulting in TgElk brain PrP^res^ accumulation were from goats. Future studies with additional goats and sheep with scrapie will be necessary to evaluate whether prion transmission from cervids to goats, although limited, may be more efficient than cervids to sheep.

Transmission of CWD from mule deer or elk to sheep has been achieved following intracerebral inoculation (Hamir *et al.*, 2006; Mitchell *et al.*, 2015). Yet, inoculation with brain homogenate from WTD with CWD did not result in brain accumulation of PrP^res^ during primary passage in ovinized mice (Tg338). One factor that may have contributed to the lack of transmission could be varying levels of PrP^Sc^ and PrP^CWD^ between brain homogenates from animals with scrapie and CWD, respectively. Laboratory biochemical assays such as WB or EIA do not correlate with infectious titres, but it has been shown that brain homogenates with higher prion levels as detected by EIA tend to also have higher infectious titres in mouse bioassay (Gonzalez *et al.*, 2012). All homogenates used in this study were prepared from cases with clinical disease at the time of cull. While a complete titration for infectivity by mouse bioassay was not performed with these samples, varying levels of PrP^Sc^ and PrP^CWD ^were detected by EIA of an endpoint dilution series for each homogenate. Several homogenates (G3953, WTD804 and CFIA113) demonstrated high PrP^Sc or CWD^ levels relative to the others by EIA, yet infectivity was primarily observed with G3953 in Tg338 (100 % attack rate at 143–146 days p.i.) and with WTD804 in TgElk (100 % attack rate at 113–128 days p.i.). Interestingly, CWD prions after primary passage through sheep (CFIA113) that appeared to have high PrP^CWD ^levels by EIA transmitted to both Tg338 and TgElk with near 100 % attack rates but cull was near or in the endpoint period. Although EIA suggests there are similar levels of PrP^Sc ^and PrP^CWD ^in these three homogenates, infectious titre or other donor or recipient mouse factors may have contributed to the variation in transmission that was observed. One factor known to contribute to scrapie transmission is *PRNP* genotype. All sheep used in the current study were of a scrapie-susceptible genotype (alanine at codon 136 and glutamine at codon 171), whereas the goats represented several different *PRNP* genotypes (previously described in O'Rourke *et al.*, 2011, 2012). There was no clear association between donor *PRNP* genotype and transmission to Tg338 or TgElk mice. It is unknown if genotype plays a role in susceptibility of sheep and goats to CWD prions, but observations by Hamir and colleagues suggest that it may (Hamir *et al.*, 2006). Additional studies will be needed to fully determine the role of *PRNP* genotype in the transmission of CWD to small ruminants.

In closing, primary passage bioassay into Tg338 (ovine *PRNP*) and TgElk (elk *PRNP*) mice produced unique transmission profiles for the isolates of CWD and scrapie prions used in this study. These transmission observations also mimicked previous research experiences of limited interspecies transmission in the natural host species. The data suggest that these two transgenic mouse models may prove to be generally robust for detecting instances of interspecies transmission of CWD to small ruminants. Additional testing of other prion isolates and characterized strains may help further establish a discriminatory mouse model using Tg338 and TgElk. Furthermore, future studies on the subpassage of the homogenates described here may help determine the potential for phenotype adaptation of CWD prions in small ruminants and aid in further strain characterization.

## Methods

### Animals

Transgenic mice and goats or sheep with naturally acquired scrapie described in this study were maintained and procedures performed in accordance with use approved by Washington State University and University of Washington Institutional Animal Care and Use Committees. Breeding pairs of ovinized mice (strain Tg338; PrP^C^ with the 136V, 154R, 171Q allele) (Laude *et al.*, 2002; Vilotte *et al.*, 2001) were kindly provided by Dr Hubert Laude (Institut National de la Recherche Agronomique, France) and held initially in a breeding colony at the University of Washington and subsequently in a breeding colony at Washington State University. Breeding pairs of cervidized mice (strain TgElk; PrP^C^ with the 132M allele) (LaFauci *et al.*, 2006) were kindly provided by Dr Robert Rowher (Research Foundation for Mental Hygiene, Inc.; Menands, NY, USA) and held in a breeding colony at Washington State University. Both lines are homozygous for the transgene with Tg338 expressing PrP^C^_VRQ_ at 8–12-fold that of sheep brain (Douet *et al.*, 2014; Laude *et al.*, 2002), whereas TgElk express PrP^C^_132M_ at 2.5-fold relative to conventional mouse (LaFauci *et al.*, 2006). Presence of the transgene was confirmed by DNA sequence analysis of tail snips and/or ear punches.

Sheep (S3178) and goats (G3538, G3558, G3953 and G30-75) with naturally acquired scrapie were referred by USDA Animal and Plant Health Inspection Service (APHIS) and are described in detail elsewhere (O'Rourke *et al.*, 2011, 2012). WTD with naturally acquired CWD were identified during CWD surveillance and tissues collected by the Nebraska Game and Parks Commission as detailed in a previous study (O'Rourke *et al.*, 2004).

The generation of sheep-passaged-CWD was conducted at the Canadian Food Inspection Agency, Ottawa Laboratory Fallowfield in accordance with Canadian Council on Animal Care guidelines under a protocol approved and monitored by the local Animal Care Committee. Under general anaesthesia, two 8-month-old ewes (CFIA113 and CFIA122) were intracerebrally inoculated with 1 ml of a 10 % (w/v in sterile normal saline) homogenate pooled from the brain and lymphoid tissues of 12 elks with CWD (CFIA Elk; all wild-type *PRNP* 132MM). Following a 1 cm skin incision, a 1 mm hole was trephined through the calvarium near the midline intersection of the parietal and frontal bones. Using a 9 cm long, 22 gauge needle the inoculum was slowly injected while withdrawing the needle from the midbrain. Beginning around 27 months post-inoculation (p.i.), both sheep began showing subtle clinical signs consistent with prion disease including ataxia, teeth grinding and progressive recumbency. At 28 months p.i. within 10 days of each other, both sheep were euthanized by barbiturate overdose and brain material was confirmed positive for pathological PrP by commercial ELISA (Bio-Rad TeSeE ELISA), WB and immunohistochemistry using methods previously described (Balachandran *et al.*, 2010; Mitchell *et al.*, 2015).

### Mouse bioassay

All inocula for mouse bioassay were prepared as previously described (O'Rourke *et al.*, 2012). Briefly, brain tissues were prepared as 10 % (w/v) homogenates in normal saline and assessed for bacterial contamination prior to the addition of gentamycin at a final concentration of 100 µg ml^−1^. Homogenates that remained positive for bacterial growth after gentamycin treatment were heat treated (80 °C for 15 min, 37 °C for 60 min and 80 °C for 15 min) and reassessed for bacterial contamination. Inoculations were only performed with homogenates cleared of bacterial contamination. PrP^Sc^ and PrP^CWD^ levels were assessed in all homogenates by EIA (HerdChek® CWD Ag Test; IDEXX Laboratories). Intracerebral inoculations were performed as described by O’Rourke *et al*. (2012). Mice were monitored and assessed for the appearance of clinical signs consistent with TSE disease (e.g. weight loss, lethargy and kyphosis). Mice were culled at clinical disease (clinical signs observed in two consecutive weeks or two out of three consecutive weeks) or within a predetermined endpoint period to limit losses due to intercurrent health issues (Bruce *et al.*, 2004). Brain was collected at euthanasia and frozen at −20 °C.

### Survival analysis

Survival time following intracerebral inoculation was defined as the number of days p.i. to the time of sacrifice due to clinical signs consistent with TSE disease in transgenic mice (Bruce *et al.*, 2004; Di Bari *et al.*, 2012). Recipient mice terminated for reasons other than clinical disease (intercurrent health issues or predetermined endpoint) were considered censored data. Mice removed from the study due to unforeseen circumstances (death within 1 week of inoculation, cannibalization by cage mate, cannibalization of a cage mate more than 30 days prior to cull or a facility issue) were not included in the calculation of survival times or shown in the graphs. The survival function of each inoculum was determined using the Kaplan–Meier product limit estimation method (LIFETEST procedure, SAS for Windows version 9.3; SAS Institute Inc.). Median survival times with 95 % confidence limits were determined by the log (−log) method.

### Western blot analysis

#### Standard Western blot (direct lysis WB).

Detection of PrP^res^ by WB was performed as previously described (O'Rourke *et al.*, 2011, 2012). Briefly, 10 % brain homogenates in PBS were diluted by adding an equal volume of 2× lysis buffer (20 mM Tris-HCl pH 7.5; 1 % NP-40, 1 % sodium deoxycholate) and incubated with proteinase K (PK). Goat, sheep, WTD and TgElk brain homogenates were incubated with 50 µg ml^−1^ PK, whereas Tg338 brain homogenates were incubated with 100  µg ml^−1^ PK for 60 min at 37 °C. Sample loading buffer (2× NuPAGE LDS Sample Buffer, Invitrogen) was added prior to electrophoresis through 12 % Bis-Tris protein gels (Invitrogen) followed by transfer of proteins to PVDF membranes. Membranes were blocked with Blocker Casein (Pierce/Thermo Scientific) prior to incubation with the anti-prion antibody F99/97.6.1 (3.5 µg ml^−1^) (O'Rourke *et al.*, 2000) and HRP-conjugated goat anti-mouse IgG_1_ (1 : 5000; Southern Biotechnology). Chemiluminescent signal of bound antibody (Amersham ECL; GE Healthcare) was captured on film (Carestream Health BioMax™ Light Film; Fisher Scientific).

#### Sodium-phosphotungstic acid Western blot (PTA-WB).

Mouse brain homogenates negative by direct lysis WB were further tested by PTA-enriched WB analysis (Spraker *et al.*, 2004; Wadsworth *et al.*, 2001). Briefly, brain homogenate was mixed 1 : 1 with 4 % (w/v) Sarkosyl and incubated at 37 °C for 15 min followed by treatment with 100  µg ml^−1^ DNase at 37 °C for 45 min. Samples were centrifuged at ~1300 ***g*** for 5 min prior to treatment of supernatants with 100  µg ml^−1^ PK at 37 °C for 60 min. Sodium-PTA (4 % w/v Na-PTA in 170 mM MgCl_2_, pH 7.4) was added to yield a final concentration of 0.3 % Na-PTA. Following incubation at 37 °C for 60 min, precipitated PrP^res^ was pelleted by centrifugation (~20 500 ***g*** for 30 min) and the final pellet dissolved in ultrapure water prior to the addition of sample loading buffer, electrophoresis, protein transfer to PVDF membrane and detection as described above.

### Molecular mass and glycoform analyses

For molecular mass and glycoform analyses of PrP^res^ in homogenate samples, WB films were scanned and saved in tiff file format with an image analyser (Alpha Innotech). Relative molecular mass of di-, mono- and unglycosylated PrP^res^ was estimated based on migration of a protein standard (MagicMark™ XP Western Protein Standard; Invitrogen) included on each blot. Statistical analyses were performed on the molecular mass of unglycosylated PrP^res^ of three or four replicate measurements for goat, sheep, deer and elk homogenate samples. The fixed effect of prion disease type was analysed using a general linear mixed model (PROC GLIMMIX; SAS 9.3; SAS Institute Inc.). Prion disease types that were considered included WTD with naturally acquired CWD, elk with naturally acquired CWD, sheep with naturally acquired scrapie, goats with naturally acquired scrapie, and sheep with experimental CWD derived from elk (sheep-passaged-CWD). The model included a random effects term for WB as each blot did not include all homogenates. Post-hoc comparison of fixed effect levels was by least squares means using stepdown Bonferroni adjustments; these options correspond to the methods of Holm (1979) for conducting sequentially rejective multiple comparisons. In addition, the Kenward and Roger option (kr) was selected for computing denominator degrees of freedom for tests of the fixed main effect and post-hoc comparisons. Holm-adjusted *P *values (*P*) were considered significant when <0.05.

Densities of di-, mono- and unglycosylated PrP^res^ were estimated using AlphaEaseFC software (Alpha Innotech). The proportional composition of the three major PrP^res^ bands is graphically presented on a three-axis plot generated using the ternary plot feature of JMP software (version 11; SAS Institute Inc.).
